# Clinical Efficacy of Adjuvant Chemotherapy in Advanced Upper Tract Urothelial Carcinoma (pT3-T4): Real-World Data from the Taiwan Upper Tract Urothelial Carcinoma Collaboration Group

**DOI:** 10.3390/jpm12020226

**Published:** 2022-02-06

**Authors:** Chung-Yu Lin, Han-Yu Weng, Ta-Yao Tai, Hsi-Chin Wu, Wen-Chi Chen, Chung-Hsin Chen, Chao-Yuan Huang, Chi-Wen Lo, Chih-Chin Yu, Chung-You Tsai, Wei-Che Wu, Yuan-Hong Jiang, Yu-Khun Lee, Thomas Y. Hsueh, Allen W. Chiu, Bing-Juin Chiang, Hsu-Che Huang, I-Hsuan Alan Chen, Yung-Tai Chen, Wei-Yu Lin, Chia-Chang Wu, Yao-Chou Tsai, Hsiang-Ying Lee, Wei-Ming Li

**Affiliations:** 1 Department of Urology, Kaohsiung Medical University Hospital, Kaohsiung 807, Taiwan; 1060489@kmuh.org.tw (C.-Y.L.); ashum1009@hotmail.com (H.-Y.L.); 2 Department of Urology, National Cheng Kung University Hospital, College of Medicine, National Cheng Kung University, Tainan 704, Taiwan; hoogagayu2@hotmail.com (H.-Y.W.); bigbite1986@gmail.com (T.-Y.T.); 3 Department of Urology, China Medical University Hospital, Taichung 404, Taiwan; wuhc@mail.cmuh.org.tw (H.-C.W.); d0283@mail.cmuh.org.tw (W.-C.C.); 4 School of Medicine, China Medical University, Taichung 404, Taiwan; 5 Department of Urology, China Medical University Beigang Hospital, Yunlin 651, Taiwan; 6 Graduate Institute of Integrated Medicine, College of Chinese Medicine, China Medical University, Taichung 404, Taiwan; 7 Department of Urology, National Taiwan University Hospital, College of Medicine, National Taiwan University, Taipei 100, Taiwan; mufasachen@gmail.com (C.-H.C.); cyh540909@gmail.com (C.-Y.H.); 8 Division of Urology, Department of Surgery, Taipei Tzu Chi Hospital, The Buddhist Medical Foundation, New Taipei City 231, Taiwan; chiwenlo0216@gmail.com (C.-W.L.); b91401049@gmail.com (C.-C.Y.); 9 School of Medicine, Buddhist Tzu Chi University, Hualien 970, Taiwan; 10 Division of Urology, Department of Surgery, Far Eastern Memorial Hospital, New Taipei City 220, Taiwan; pgtsai@gmail.com (C.-Y.T.); wuweiche@gmail.com (W.-C.W.); 11 Department of Healthcare Information and Management, Ming Chuan University, Taipei 111, Taiwan; 12 Institute of Biomedical Engineering, National Taiwan University, Taipei 106, Taiwan; 13 Department of Urology, Hualien Tzu Chi Hospital, Buddhist Tzu Chi Medical Foundation and Tzu Chi University, Hualien 970, Taiwan; redeemerhd@gmail.com (Y.-H.J.); leeyukhun@gmail.com (Y.-K.L.); 14 Division of Urology, Department of Surgery, Taipei City Hospital Renai Branch, Taipei 106, Taiwan; tyjhsueh@gmail.com; 15 Department of Urology, School of Medicine, National Yang Ming Chiao Tung University, Taipei 112, Taiwan; 16 College of Medicine, National Yang Ming Chiao Tung University, Taipei 112, Taiwan; whchiu1216@gmail.com; 17 College of Medicine, Fu-Jen Catholic University, New Taipei City 242, Taiwan; bingjuinchiang@gmail.com; 18 Division of Urology, Department of Surgery, Cardinal Tien Hospital, New Taipei City 231, Taiwan; huangshuche@gmail.com; 19 Department of Life Science, College of Science, National Taiwan Normal University, Taipei 116, Taiwan; 20 Division of Urology, Department of Surgery, Kaohsiung Veterans General Hospital, Kaohsiung 813, Taiwan; alan_aries@yahoo.com.tw; 21 Department of Urology, Taiwan Adventist Hospital, Taipei 105, Taiwan; urochen831@gmail.com; 22 Division of Urology, Department of Surgery, Chang Gung Memorial Hospital, Chia-Yi 613, Taiwan; lwy0912@yahoo.com; 23 Chang Gung University of Science and Technology, Chia-Yi 613, Taiwan; 24 Department of Medicine, Chang Gung University, Taoyuan 333, Taiwan; 25 Department of Urology, Shuang Ho Hospital, Taipei Medical University, New Taipei City 235, Taiwan; charleswjj@yahoo.com.tw; 26 Department of Urology, School of Medicine, College of Medicine, Taipei Medical University, Taipei 110, Taiwan; tsai1970523@yahoo.com.tw; 27 TMU Research Center of Urology and Kidney (TMU-RCUK), Taipei Medical University, Taipei 110, Taiwan; 28 Department of Urology, Taipei Medical University Hospital, Taipei Medical University, Taipei 110, Taiwan; 29 Department of Urology, School of Medicine, College of Medicine, Kaohsiung Medical University, Kaohsiung 807, Taiwan; 30 Center for Liquid Biopsy and Cohort Research, Kaohsiung Medical University, Kaohsiung 807, Taiwan; 31 Department of Urology, Ministry of Health and Welfare, Pingtung Hospital, Pingtung 900, Taiwan

**Keywords:** upper tract urothelial carcinoma, chemotherapy, prognosis

## Abstract

The clinical efficacy of adjuvant chemotherapy in upper tract urothelial carcinoma (UTUC) is unclear. We aimed to assess the therapeutic outcomes of adjuvant chemotherapy in patients with advanced UTUC (pT3-T4) after radical nephroureterectomy (RNU). We retrospectively reviewed the data of 2108 patients from the Taiwan UTUC Collaboration Group between 1988 and 2018. Comprehensive clinical features, pathological characteristics, and survival outcomes were recorded. Univariate and multivariate Cox proportional hazards models were used to evaluate overall survival (OS), cancer-specific survival (CSS), and disease-free survival (DFS). Of the 533 patients with advanced UTUC included, 161 (30.2%) received adjuvant chemotherapy. In the multivariate analysis, adjuvant chemotherapy was significantly associated with a reduced risk of overall death (hazard ratio (HR), 0.599; 95% confidence interval (CI), 0.419–0.857; *p* = 0.005), cancer-specific mortality (HR, 0.598; 95% CI, 0.391–0.914; *p* = 0.018), and cancer recurrence (HR, 0.456; 95% CI, 0.310–0.673; *p* < 0.001). The Kaplan–Meier survival analysis revealed that patients receiving adjuvant chemotherapy had significantly better five-year OS (64% vs. 50%, *p* = 0.002), CSS (70% vs. 62%, *p* = 0.043), and DFS (60% vs. 48%, *p* = 0.002) rates compared to those who did not receive adjuvant chemotherapy. In conclusion, adjuvant chemotherapy after RNU had significant therapeutic benefits on OS, CSS, and DFS in advanced UTUC.

## 1. Introduction

Upper tract urothelial carcinoma (UTUC) is a relatively rare tumor that accounts for 10% of all renal tumors and 5% of urothelial malignancies overall, with an estimated annual incidence of almost two cases per 100,000 inhabitants in Western countries [1,2]. However, the epidemiology and clinical presentation of UTUCs in Taiwan are quite different from those in Western populations [3,4,5]. In Taiwan, the incidence rate of UTUC is much higher than that worldwide and in Western populations, and the prevalence of UTUC is as high as 30% of all urothelial carcinomas (UCs) [3,4,5]. Several risk factors associated with UCs, such as exposure to arsenic, toxic chemicals, aristolochic acid, and cigarette smoking, have been reported in previous studies [6,7]. Radical nephroureterectomy (RNU) with bladder cuff excision is regarded as the standard therapy for local UTUC, but monotherapy with surgical intervention is considered to be associated with relatively high recurrence rates and low survival rates in the advanced pathological stage [2,4,5,8]. The five-year overall survival rate is less than 50% in patients with T2–3 and less than 10% in patients with T4 [2,9].

In the current study, UC is chemosensitive cancer, and the effectiveness of chemotherapy in treating urinary bladder UC has been well established [2,10,11]. Thus, postoperative adjuvant chemotherapy has been suggested to patients with advanced-stage UC, such as those with ≥ pathological T3 or node-positive disease [2,10,11]. However, there is less strong evidence for adjuvant chemotherapy followed by RNU in patients with UTUC. Although the current guidelines suggest that cisplatin-based perioperative chemotherapy can provide a survival benefit for patients with advanced stage, the current evidence and practice are still mainly dependent on data from bladder cancer due to the rarity of UTUC worldwide [2,11].

Therefore, this study aimed to investigate the current role of postoperative adjuvant chemotherapy in patients with advanced UTUC (pathological T3–T4) in a large multicenter cohort from Taiwan and to set a benchmark for comparison of future novel trials.

## 2. Materials and Methods

### 2.1. Patient Population

Taiwan UTUC Collaboration Group conducted this nationwide study. We included 14 participating Taiwan hospitals (Kaohsiung Medical University Hospital, Taipei Tzu Chi Hospital, Hualien Tzu Chi Hospital, Chang Gung Memorial Hospital, Chiayi, Kaohsiung Veterans General Hospital, China Medical University Hospital, Taipei City Hospital, Taiwan Adventist Hospital, National Taiwan University Hospital, Taipei Medical University Shuang Ho Hospital, Cardinal Tien Hospital, National Cheng Kung University Hospital, Taipei Medical University Hospital, and Far Eastern Memorial Hospital). This study was approved by the institutional review board (KMUHIRB-E(I)-20190107). We retrospectively reviewed the data of 2108 patients diagnosed with UTUC between 1988 and 2018. Among the 2108 patients, 1524 were excluded from this study, 293 did not undergo RNU, 1201 had early-stage UTUC (pathological Ta-T2), seven were not UC, 16 were treated with neoadjuvant chemotherapy, 41 were with positive surgical margin, and 19 were followed up <1 month. The remaining 584 patients with advanced disease (pathological T3–T4) who underwent RNU were included for further analysis.

The following characteristics were included: sex, age, body mass index (BMI), Eastern Cooperative Oncology Group (ECOG) performance status, American Society of Anesthesiologists (ASA) physical status classification system, smoking, tumor size, tumor number, tumor location, tumor grade, tumor stage, concurrent carcinoma in situ (CIS), lymphovascular invasion (LVI), histological variants, lymph node status, surgical margin, and adjuvant chemotherapy. Tumor staging was defined according to the 2010 American Joint Committee on Cancer TNM staging system, and tumor grade was determined based on the 2004 WHO classification. Adjuvant chemotherapy was defined as chemotherapy administered within three months after RNU. Most patients received platinum-based chemotherapy, including methotrexate, vinblastine, doxorubicin, cisplatin/carboplatin (MVAC), or gemcitabine plus cisplatin/carboplatin. After RNU, patients received regular follow-up programs, including physical, laboratory, and radiological examinations, according to standard guidelines. UTUC recurrence was defined as local recurrence at the tumor bed, regional lymph node metastasis, or distant organ metastasis. The cause of death was determined according to death certificates.

### 2.2. Statistical Analysis

To compare the clinicopathological characteristics of the two groups (with vs. without adjuvant chemotherapy), a Student’s *t*-test and Pearson’s chi-square test were used for continuous and categorical variables, respectively. We used the Kaplan–Meier survival analysis to estimate the clinical outcomes, including overall survival (OS), cancer-specific survival (CSS), and disease-free survival (DFS). Univariate and multivariate Cox proportional hazard regression models with Enter method were used to identify independent prognostic factors of clinical outcomes (OS, CSS, and DFS). We used the landmark method to minimize immortal time bias. Patients with survival > 1 month were only included to account for immortal time bias in patients who were not able to receive adjuvant chemotherapy and to control the acute surgical complications.

All statistical analyses were conducted using SPSS version 26 (IBM, Armonk, NY, USA), and *p*-values of <0.05 were considered statistically significant.

## 3. Results

### 3.1. Patient Characteristics

This study observed 533 patients diagnosed with advanced UTUC (pT3–T4) after standard treatment with RUN. A total of 247 males and 286 females participated in the study. The adjuvant chemotherapy group consisted of 161 (30.2%) patients with UTUC who received chemotherapy followed by RNU. The non-adjuvant chemotherapy group comprised 372 (69.8%) patients treated with initial surgical management that did not undergo adjuvant chemotherapy. The clinical characteristics of the patients are summarized in Table 1. The mean age of the study population was 68.6 years. The mean length of follow-up was 54.1 months. In all, 472 patients (88.6%) were diagnosed with pT3 disease, and 61 (11.4%) were diagnosed with pT4 disease. Regarding kidney function, the mean eGFR was 50.4 mL/min per 1.73 m^2^, and 35% patients had chronic kidney disease (eGFR < 60 mL/min). After RNU, we found a mean difference between preoperative and postoperative eGFR of 10.1 mL/min per 1.73 m^2^ (*p* < 0.001). The median follow-up period was 46.8 months in the adjuvant chemotherapy group and significantly longer than in the non-adjuvant chemotherapy group at 29 months (*p* = 0.001). The patients receiving adjuvant chemotherapy were younger (64.3 ± 9.9 vs. 70.5 ± 11.2 years, *p* < 0.001) and had better ASA physical status (*p* = 0.009) and lymph node metastasis (13.7% vs. 5.4%, *p* = 0.011) than those without chemotherapy administration. No significant differences were found in terms of sex, BMI, ECOG performance status, and tumor presentation characteristics, including tumor location, tumor size, histological variants of the tumor, tumor grade, presence of CIS, LVI, and pathological stage.

### 3.2. Clinical Outcomes

Univariate analyses for OS, CSS, and DFS rates are summarized in Table 2. We found that adjuvant chemotherapy, tumor size, tumor grade, multiplicity of the tumor, and pathological stage, including T and N stage, were significantly associated with OS, CSS, and DFS. Age and lymphovascular invasion were significant prognostic factors for OS and CSS but not for DFS. Smoking history was significantly correlated with CSS but not DFS and OS. Different chemotherapy regimen was not a significant predictor for OS, CSS, and DFS.

In multivariable analysis, adjuvant chemotherapy was demonstrated to be a significant independent prognostic factor of OS (hazard ratio (HR), 0.599; 95% confidence interval (CI), 0.419–0.857; *p* = 0.005), CSS (HR, 0.598; 95% CI, 0.391–0.914; *p* = 0.018), and DFS (HR, 0.456; 95% CI, 0.310–0.673; *p* < 0.001) in pT3–T4 UTUC patients after RUN (Table 3). We also found that high tumor grade, high pathological tumor stage, and lymph node metastasis were significantly independently associated with worse OS, CSS, and DFS. Age was a significant prognostic factor for OS but not for CSS and DFS.

**Figure 1:**
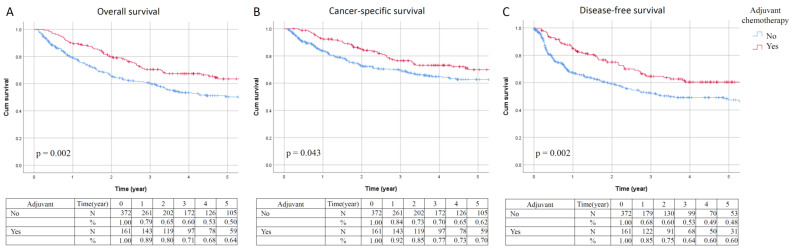
Kaplan–Meier estimates of overall survival (**A**), cancer-specific survival (**B**), and disease-free survival (**C**).

**Figure 2:**
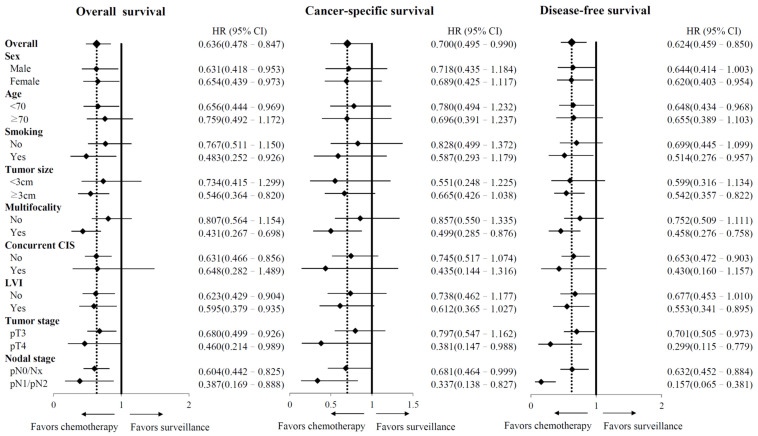
Subgroup analysis of overall survival, cancer-specific survival, and disease-free survival according to baseline characteristics. HR—hazard ratio; CI—confidence interval; CIS—carcinoma in situ; LVI—lymphovascular invasion.

The Kaplan–Meier survival analysis (Figure 1) revealed that patients with advanced UTUC who underwent adjuvant chemotherapy had significantly better five-year OS (64% vs. 50%, *p* = 0.002), CSS (70% vs. 62%, *p* = 0.043), and DFS (60% vs. 48%, *p* = 0.002) rates than those who did not receive adjuvant chemotherapy. In competing risk analysis, adjuvant chemotherapy improved CSS (sHR, 0.735; 95% CI, 0.400–1.070; *p* = 0.073) and DFS (sHR, 0.657; 95% CI, 0.367–0.947; *p* = 0.004) (Table S1). Overall, the observed treatment effect on OS, CSS, and DFS was consistent across subgroups (Figure 2).

## 4. Discussion

The standard procedure for treating UTUC is RNU regardless of the tumor location and excellent oncologic outcomes and lower recurrence rates in T1 and T2 stage disease have been reported [2,4,5]. In patients with advanced UTUC, the disease has a high systemic recurrence rate and poor prognosis despite radical surgery [2,4,5]. Therefore, adjuvant chemotherapy is considered a reasonable strategy to improve oncologic outcomes and is suggested in the current guidelines [2,11]. Nevertheless, there is currently a lack of data concerning the survival benefit of adjuvant chemotherapy in patients with advanced UTUC, and the data remain controversial and conflicting because of the rarity of UTUC [9]. To address these limitations, we performed an analysis of patients with UTUC from 14 participating Taiwan hospitals, including almost 584 patients with pT3/T4 and without distant metastasis who received either adjuvant chemotherapy or surveillance, respectively.

Previous studies have supported the survival benefits of adjuvant chemotherapy after surgical intervention. In 2020, the first phase III randomized trial (POUT) revealed the benefit of adjuvant systemic chemotherapy after RNU [12]. In 2017, Nakagawa et al. reported that adjuvant chemotherapy improved CSS in a cohort of 109 patients with advanced-stage UTUC after RUN [13]. In 2019, Huang et al. also demonstrated the therapeutic benefit of adjuvant chemotherapy in stage II-IV local UTUC [14].

In the POUT trial [12], the patients were recorded from 71 National Health Service (NHS) hospitals in the UK. Although the race distribution of patients was not mentioned in the POUT study, the underrepresentation of Asian patients in the POUT trial was possible. To the best of our acknowledge, the incidence and presentation of UTUC in the Asian population were different from those in the Western population. We believed our study made up for the lack of evidence and evaluated the effect of adjuvant chemotherapy for East Asian UTUCs patients.

Real-world patients may have poorer performance status and compliance and may include higher proportions of elderly patients. These patients may be excluded from RCT but should not be neglected in our clinical practice. We thought our real-world data were essential and could reflect clinical experience, especially in Asian groups. Our study included patients with high comorbidity (ASA3 and ECOG ≥ 2). We performed subgroup analysis and found adjuvant chemotherapy significantly improved OS, CSS, and DFS in this patient population (Figures S1 and S2). High comorbidities in UTUC may result in competing mortality. We excluded patients who died due to non-cancer before tumor recurrence to minimize competing bias. The results showed that adjuvant chemotherapy was significantly associated with better CSS and DFS (Figure S3). In addition, we also had large case numbers and a long-term follow-up period to strengthen our results. In contrast to our results, Necchi et al. reported that adjuvant chemotherapy was not associated with any survival benefit in high-risk (≥pT2 and/or pN1-3) UTUC patients compared to observation following RNU in a retrospective study of 1544 patients [15]. Vassilakopoulou et al. observed no benefits of postoperative chemotherapy in patients with high-risk UTUC [16]. These conflicting results between our study and other studies may have been because both non-cisplatin-based and cisplatin-based adjuvant chemotherapy were included in the study by Necchi et al., and there were unknown regimens in some patients (115/312, 35.8%) [15]. However, most patients in the current study underwent platinum-based adjuvant chemotherapy, including cisplatin and carboplatin. Chemotherapy regimens may have contributed to the differences in results between these studies.

The other possible explanation is the population with metastatic disease in these studies. In our retrospective study, only advanced patients were included. Nevertheless, several other studies enrolled patients with distant metastasis [15,16]. Patients with metastatic disease who underwent salvage adjuvant chemotherapy had equivocal benefits in terms of survival outcomes [17]. The benefit may be biased because younger and healthier patients tend to receive chemotherapy, and it may be that the apparent survival time was prolonged. In our opinion, the negative results of these studies were influenced by enrolling patients with metastatic disease that shortened the average survival time and contributed to the lack of survival improvement in these studies.

Platinum-based chemotherapy was selected for adjuvant chemotherapy in most patients included in the current study. Moreover, the patients who underwent adjuvant chemotherapy were younger and had a better renal function before and after surgery than those who did not. Patients were treated with a cisplatin-based regimen if the renal function was adequate to tolerate adjuvant chemotherapy. Some patients who had declining renal function and/or decreased tolerability for adjuvant chemotherapy following RUN underwent adjuvant chemotherapy with a carboplatin-based regimen. In a previous study, carboplatin was a useful alternative solution when cisplatin was contraindicated and not at the expense of efficacy [18]. Chang et al. also supported the efficacy of adjuvant chemotherapy with carboplatin in patients with advanced UTUC [14]. In contrast, Leow et al. compared non-cisplatin-based or cisplatin-based adjuvant chemotherapy after RUN and found no survival benefit for non-cisplatin-based regimens [19].

Although the different adjuvant chemotherapy regimens were not a significant prognostic factor in our study, platinum-based chemotherapy for patients with advanced UTUC still had a better treatment effect on survival outcomes than non-platinum-based chemotherapy. Furthermore, the carboplatin-based regimen had relatively better CSS and DFS than the cisplatin-based regimen. This finding contrasts with the results of other studies. In the POUT trial, cisplatin–gemcitabine chemotherapy showed a superior tumor response rate than carboplatin–gemcitabine adjuvant chemotherapy [12]. However, patients with impaired renal function still have survival benefits from the gemcitabine–carboplatin regimen. We cannot be sure why we could not obtain the same result seen in some clinical trials. However, we believe that the following are several possibilities. First, our patients may not be as healthy as the participants in these trials. Good performance status is always an entry criterion for most trials. Second, cisplatin treatment, which follows a strict protocol in prospective trials, is better than clinical practice in reducing mortality. In our opinion, nephrotoxicity of cisplatin may be associated with more complications, such as acute kidney injury, electrolyte imbalance, or erythropoietin deficiency, than carboplatin, which influences the survival outcome in patients with advanced UTUC in real-world practice [20]. The effectiveness of adjuvant chemotherapy with carboplatin-based regimens is still controversial in UTUC. However, our study may provide evidence to support the therapeutic role of carboplatin-based adjuvant chemotherapy in patients with contraindications to cisplatin-based regimens because of impaired renal function.

In our study, the patients who underwent adjuvant chemotherapy had more aggressive pathological features with advanced lymphadenopathy (>pN0). At the time of data collection, lymph node dissection (LND) was not a part of standard care because there were little data to support this procedure strongly [2,21]. Nodal dissection has a controversial therapeutic role, and the procedure is performed if nodal metastasis is defined in the clinical image. Dominguez-Escrig et al., Don et al., and Zhai et al. reported that LND had a survival benefit in patients with high stage (≥pT2) UTUC and reduced the risk of local recurrence [22,23,24]. Guo et al. demonstrated that LND during RUN provides more accurate pathological staging and prediction of prognosis [25]. In our opinion, LND could help physicians identify candidates for adjuvant chemotherapy in patients with advanced UTUC, and our results also reinforced the benefit of adjuvant chemotherapy for pT3/T4N+ patients.

To our knowledge, LVI is a key and significant step in tumor spreading and metastasis and has been regarded as a prognostic factor for UTUC after RNU [2,26,27,28]. In our study, univariate analysis of survival outcomes revealed that LVI status was associated with worse OS, CSS, and DFS (*p* < 0.005). However, LVI status became a non-significant predictive factor when other variables were included in the multivariate analysis. This result is similar to that of Chen et al., who reported that LVI was not associated with survival outcomes of UTUC with the pT3 stage [29]. In our opinion, T3 or T4 disease means that tumor cells invade peripheral soft tissue and show a more predominant influence than LVI status on survival outcome. The impact of LVI status is minimal in patients with T3/T4 stage and no suitable prognostic factor for survival outcome.

The present study had several limitations. First, this is a retrospective observational study with unavoidable shortcomings, such as inconsistent data collection during the study period, and selection. Evolution of medical care, technology, chemotherapy protocols, and differences in the surgeon experience may also be a source of bias, although the clinical outcomes were not significantly associated with the year of UTUC diagnosis (Figure S4). Second, although the was no significant difference in the patient’s general status, more healthy patients were in the adjuvant chemotherapy group. The healthier patient group may contribute to a better clinical outcome and weaken the conclusion of our study. Third, we only perform lymph node dissection in patients with lymph-node involvement identified on preoperative imaging or during surgery. The actually node-positive UTUC disease cannot be completely studied. Last, the patients who received platinum-based chemotherapy were all included regardless of cisplatin or carboplatin in our study, and the cut-off value of the renal function to carboplatin-based regimen might be different in each hospital. A meta-analysis revealed superior tumor response rates in patients with advanced UC treated with cisplatin than those treated with carboplatin [30]. Therefore, the efficacy of adjuvant chemotherapy following RUN may be underestimated. Finally, although our retrospective UTUC cohort was quite large, further prospective, randomized, and multi-institutional studies may be necessary to confirm our results.

## 5. Conclusions

We demonstrated a significant improvement in OS, CSS, and DFS with adjuvant chemotherapy after RNU for patients with advanced UTUC in this large observational study. These results support the use of adjuvant chemotherapy and may assist physicians in patient consultation and clinical decision making.

## Figures and Tables

**Table 1:** Patients’ demographic and clinicopathological characteristics.

Variables	Adjuvant Chemotherapy (*n* = 161)		No Adjuvant Chemotherapy (*n* = 372)		*p*-Value
	*n*	(%)	*n*	(%)	
Sex					
Male	80	(49.7)	167	(44.9)	0.308
Female	81	(50.3)	205	(55.1)	
Age (years)					
Mean ± SD	64.3 ± 9.9		70.5 ± 11.2		<0.001 *
Median	65.9		726		
BMI (kg/m^2^)					
Mean ± SD	24.5 ± 4.0		23.9 ± 3.8		0.386
Median	24.1		23.8		
Follow-up (months)					
Mean ± SE	60.1 ± 7.6		51.2 ± 7.8		<0.001 *
Median	46.8		29.0		
ASA					
1	2	(2.0)	4	(2.4)	0.009 *
2	51	(50.0)	50	(29.8)	
3	49	(48.0)	113	(67.3)	
4	0	(0.0)	1	(0.6)	
ECOG					
0	58	(46.8)	98	(41.0)	0.293
1	55	(44.4)	102	(42.7)	
2	10	(8.1)	31	(13.0)	
3	1	(0.8)	4	(1.7)	
4	0	(0.0)	4	(1.7)	
Smoking					
No	85	(72.0)	182	(71.7)	0.940
Yes	33	(28.0)	72	(28.3)	
Tumor location					
Renal pelvis	80	(49.7)	202	(54.3)	0.317
Ureter	50	(31.1)	92	(24.7)	
Renal pelvis + ureter	31	(19.3)	78	(21.0)	
Tumor size					
<1 cm	3	(2.3)	10	(3.2)	0.760
≥1 and <2 cm	22	(16.5)	39	(12.7)	
≥2 and <3 cm	26	(19.5)	56	(18.2)	
≥3 cm	82	(61.7)	203	(65.9)	
Histological variant					
No	135	(83.9)	322	(86.6)	0.412
Yes	26	(16.1)	50	(13.4)	
Tumor grade					
Low grade	12	(7.5)	15	(4.1)	0.098
High grade	148	(92.5)	355	(95.9)	
Multifocality					
No	102	(63.4)	234	(62.9)	0.921
Yes	59	(36.6)	138	(37.1)	
Concurrent CIS					
No	141	(87.6)	325	(87.4)	0.946
Yes	20	(12.4)	47	(12.6)	
Lymphovascular invasion					
No	99	(61.5)	245	(65.9)	0.333
Yes	62	(38.5)	127	(34.1)	
Pathological stage					
Stage III	128	(79.5)	308	(83.2)	0.301
Stage IV	33	(83.2)	62	(16.7)	
Pathological T stage					
pT3	146	(90.7)	326	(87.6)	0.310
pT4	15	(9.3)	46	(12.4)	
Pathological N stage					
pN0	38	(23.6)	74	(19.9)	0.011 *
pN1	10	(6.2)	10	(2.7)	
pN2	12	(7.5)	10	(2.7)	
pNx	98	(60.9)	272	(73.1)	

* *p* < 0.05.

**Table 2:** Univariate analyses for overall, cancer-specific, and disease-free survivals in advanced UTUC patients.

	Overall Survival			Cancer-Specific Survival			Disease-Free Survival		
Variables	HR	95% CI	*p*-Value	HR	95% CI	*p*-Value	HR	95% CI	*p*-Value
Adjuvant chemotherapy									
No	1			1			1		
Yes	0.636	0.478–0.847	0.002 *	0.700	0.495–0.990	0.044 *	0.624	0.459–0.850	0.003 *
Chemotherapy regimens									
Gemcitabine and cisplatin	1				1				1
MVAC	1.014	0.598–1.718	0.960	0.847	0.475–1.511	0.574	0.794	0.480–1.316	0.371
Carboplatin-based	0.529	0.127–2.204	0.382	0.314	0.043–2.296	0.254	0.482	0.116–1.998	0.315
Others	1.215	0.774–1.905	0.397	1.048	0.640–1.714	0.853	1.057	0.689–1.624	0.799
Sex									
Male	1			1			1		
Female	1.011	0.788–1.296	0.932	1.110	0.814–1.515	0.509	1.119	0.851–1.472	0.421
Age									
<70	1			1			1		
≥70	1.750	1.360–2.253	<0.001 *	1.407	1.031–1.921	0.031 *	1.280	0.971–1.686	0.080
BMI									
<24	1			1			1		
≥24	0.896	0.647–1.241	0.509	0.989	0.678–1.444	0.956	0.990	0.698–1.402	0.953
ASA									
1	1			1			1		
>1	2.527	0.352–18.131	0.357	1.833	0.254–13.201	0.548	1.202	0.296–4.885	0.797
ECOG									
0	1			1			1		
≥1	1.115	0.805–1.544	0.513	0.978	0.672–1.423	0.906	1.028	0.729–1.449	0.876
Smoking									
No	1			1			1		
Yes	1.181	0.849–1.643	0.323	1.482	1.005–2.186	0.047 *	1.368	0.968–1.934	0.076
Hospitals									
Medical centers	1			1			1		
Regional hospitals	1.111	0.880–1.403	0.376	1.166	0.882–1.541	0.280	1.105	0.855–1.429	0.446
Tumor location									
Renal pelvis	1			1			1		
Ureter	1.134	0.848–1.517	0.395	1.358	0.952–1.937	0.091	1.349	0.979–1.858	0.067
Renal pelvis + ureter	1.200	0.869–1.657	0.267	1.214	0.808–1.824	0.351	1.289	0.910–1.826	0.153
Tumor size									
<3 cm	1			1			1		
≥3 cm	1.478	1.082–2.018	0.014 *	1.898	1.281–2.810	0.001 *	1.607	1.154–2.240	0.005 *
Histological variants									
No	1			1			1		
Yes	1.185	0.828–1.694	0.353	1.355	0.890–2.063	0.156	1.105	0.754–1.620	0.609
Tumor grade									
Low grade	1			1			1		
High grade	2.854	1.269–6.421	0.011 *	5.804	1.438–23.414	0.013 *	4.212	1.565–11.339	0.004 *
Multifocality									
No	1			1			1		
Yes	1.367	1.062–1.758	0.015 *	1.407	1.029–1.924	0.033 *	1.390	1.053–1.836	0.020 *
Concurrent CIS									
No	1			1			1		
Yes	1.122	0.751–1.676	0.575	1.125	0.696–1.817	0.632	0.959	0.626–1.469	0.847
Lymphovascular invasion									
No	1			1			1		
Yes	1.476	1.139–1.914	0.003 *	1.672	1.223–2.285	0.001 *	1.305	0.988–1.725	0.061
Pathological T stage									
pT3	1			1			1		
pT4	2.723	1.980–3.746	<0.001 *	3.120	2.124–4.583	<0.001 *	2.294	1.567–3.357	<0.001 *
Pathological N stage									
pN0 + pNx	1			1			1		
pN1 + pN2	0.474	0.313–0.718	<0.001 *	0.389	0.246–0.617	<0.001	0.410	0.269–0.625	<0.001 *

* *p* < 0.05. HR—hazard ratio; CI—confidence interval.

**Table 3:** Multivariate analyses for overall, cancer-specific, and disease-free survivals in advanced UTUC patients.

	Overall Survival			Cancer-Specific Survival			Disease-Free Survival		
Variables	HR	95% CI	*p*-Value	HR	95% CI	*p*-Value	HR	95% CI	*p*-Value
Adjuvant chemotherapy									
No	1			1			1		
Yes	0.599	0.419–0.857	0.005 *	0.598	0.391–0.914	0.018 *	0.456	0.310–0.673	<0.001 *
Age									
<70	1			1			1		
≥70	1.727	1.272–2.346	<0.001 *	1.321	0.917–1.902	0.134	1.176	0.856–1.616	0.317
Tumor size									
<3 cm	1			1			1		
≥3 cm	1.188	0.859–1.643	0.299	1.473	0.974–2.226	0.066	1.377	0.978–1.939	0.067
Tumor grade									
Low grade	1			1			1		
High grade	2.526	1.033–6.181	0.042 *	8.184	1.139–58.824	0.037 *	3.772	1.197–11.885	0.023 *
Multifocality									
No	1			1			1		
Yes	1.274	0.951–1.707	0.104	1.375	0.966–1.957	0.077	1.415	1.043–1.920	0.026 *
Lymphovascular invasion									
No	1			1			1		
Yes	1.258	0.935–1.693	0.129	1.395	0.970–2.006	0.073	1.193	0.870–1.635	0.274
Pathological T stage									
pT3	1			1			1		
pT4	2.390	1.588–3.596	<0.001 *	2.234	1.377–3.627	0.001 *	1.654	1.033–2.650	0.036 *
Pathological N stage									
pN0 + pNx	1			1			1		
pN1 + pN2	2.234	1.397–3.572	0.001 *	2.323	1.361–3.963	0.002 *	2.888	1.755–4.752	<0.001 *

* *p* < 0.05. HR—hazard ratio; CI—confidence interval.

## Data Availability

All data generated or analyzed during this study are included in this published article.

## Supplementary Materials

The following supporting information can be downloaded at: https://www.mdpi.com/article/10.3390/jpm12020226/s1, Figure S1: Subgroup survival analysis of ASA = 3; Figure S2: Subgroup survival analysis of ECOG > 2; Figure S3: Survival analysis in our cohort excluding non-cancer mortality patients, Figure S4: Survival analysis according to year of UTUC diagnosis, Table S1: Competing risk analyses for cancer-specific and disease-free survivals in advanced UTUC patients.
