# Supplementary material for: Clinical Efficacy of Adjuvant Chemotherapy in Advanced Upper Tract Urothelial Carcinoma (pT3-T4): Real-World Data from the Taiwan Upper Tract Urothelial Carcinoma Collaboration Group
Source: J Pers Med. 2022 Feb 6;12(2):226. doi: 10.3390/jpm12020226 (PMC8877034; doi:10.3390/jpm12020226)
Supplement: Supplementary file 1 [file jpm-12-00226-s001.zip › jpm-1495908-supplementary/Table S1.pdf]

Table S1. Competing risk analyses for cancer-specific and disease-free survivals in advanced UTUC patients

	Cancer-specific survival			Disease-free survival		
Variables	sHR	95% CI	*p* value	sHR	95% CI	*p* value
Adjuvant chemotherapy						
No	1			1		
Yes	0.735	0.400–1.070	0.073	0.657	0.367–0.947	0.004*
Sex						
Male	1			1		
Female	1.079	0.810–1.348	0.577	1.143	0.829–1.457	0.404
Age						
<70	1			1		
≥70	1.288	1.016–1.560	0.069	1.235	0.919–1.551	0.192
BMI						
<24	1			1		
≥24	0.989	0.644–1.334	0.952	0.939	0.553–1.325	0.939
ASA						
1	1			1		
>1	1.200	0.177–2.223	0.800	2.032	0.148–3.916	0.460
ECOG						
0	1			1		
≥1	1.032	0.691–1.373	0.857	0.869	0.489–1.249	0.472
Smoking						
No	1			1		
Yes	0.492	0.157–0.827	0.019*	1.300	0.900–1.700	0.200
Tumor location						
Renal pelvis	1			1		
Ureter	1.163	0.994–1.332	0.080	1.116	0.924–1.308	0.268

Tumor size						
<3cm	1			1		
≥ 3cm	1.602	1.284–1.920	0.003*	1.873	1.485–2.261	0.001*
Histological variants						
No	1			1		
Yes	1.192	0.800–1.586	0.383	1.189	0.752–1.626	0.438
Tumor grade						
Low grade	1			1		
High grade	3.839	2.839–4.839	0.008*	5.502	4.106–6.898	0.016*
Multifocality						
No	1			1		
Yes	1.309	0.995–1.623	0.056	1.379	1.061–1.697	0.048*
Concurrent CIS						
No	1			1		
Yes	1.028	0.605–1.451	0.898	1.000	0.512–1.488	1.000
Lymphovascular invasion						
No	1			1		
Yes	1.484	1.210–1.758	0.005*	1.674	1.358–1.990	0.001*
Pathological T stage						
pT3	1			1		
pT4	2.401	2.003–2.799	<0.001*	3.192	2.751–3.623	<0.001*
Pathological N stage						
pN0+pNx	1			1		
pN1+pN2	2.578	2.131–3.025	<0.001*	2.445	1.951–2.939	<0.001*

*p<0.05. sHR subdistribution hazard ratio. CI–confidence interval.
