# Supplementary figures and images for: Clinical Efficacy of Adjuvant Chemotherapy in Advanced Upper Tract Urothelial Carcinoma (pT3-T4): Real-World Data from the Taiwan Upper Tract Urothelial Carcinoma Collaboration Group
Source: J Pers Med. 2022 Feb 6;12(2):226. doi: 10.3390/jpm12020226 (PMC8877034; doi:10.3390/jpm12020226)

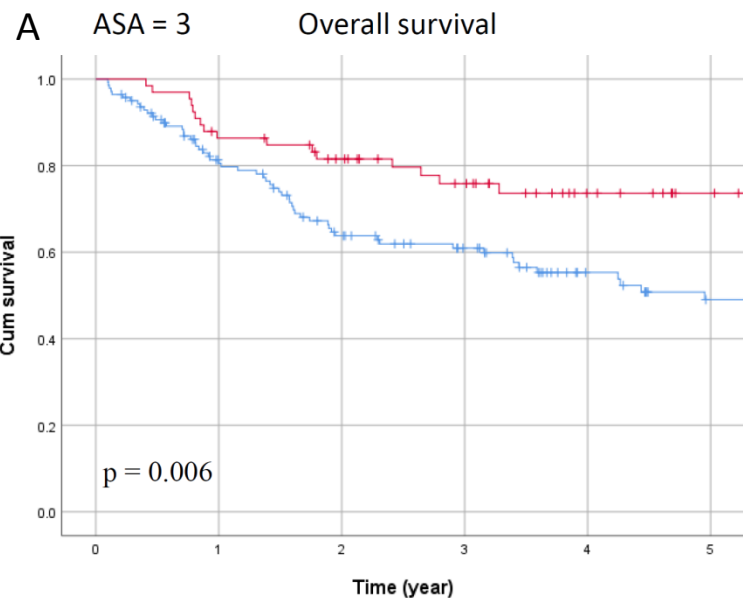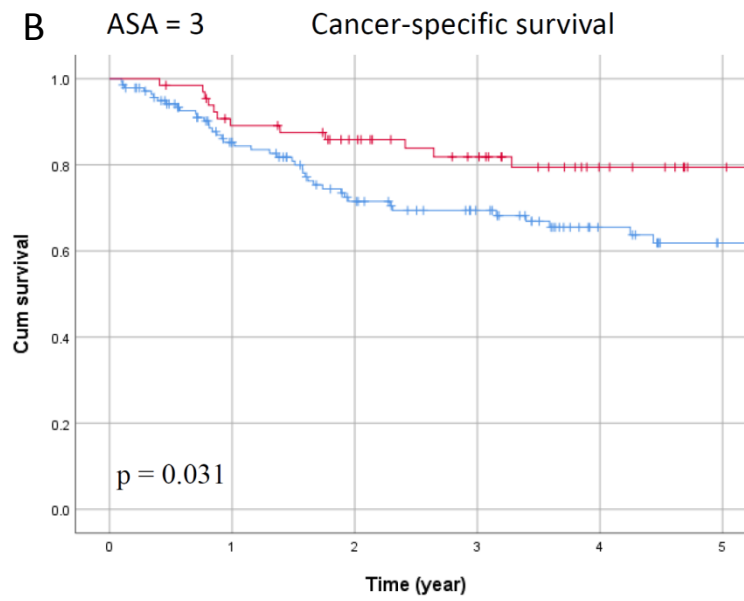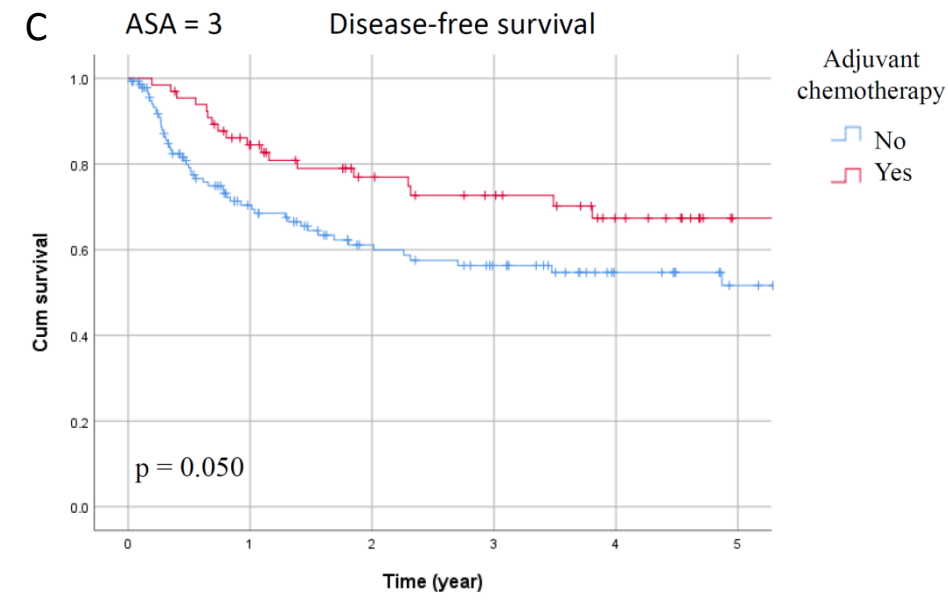

Supplement: Supplementary file 1 [file jpm-12-00226-s001.zip › jpm-1495908-supplementary/Figure S1.pdf]

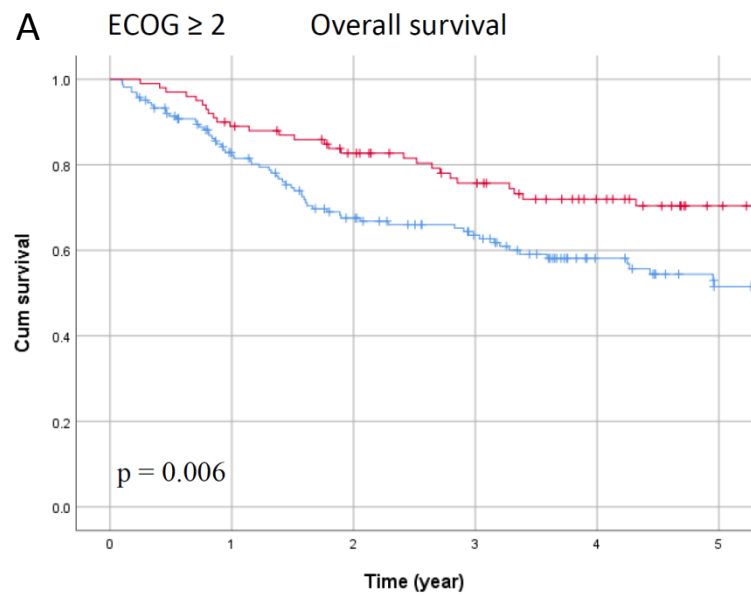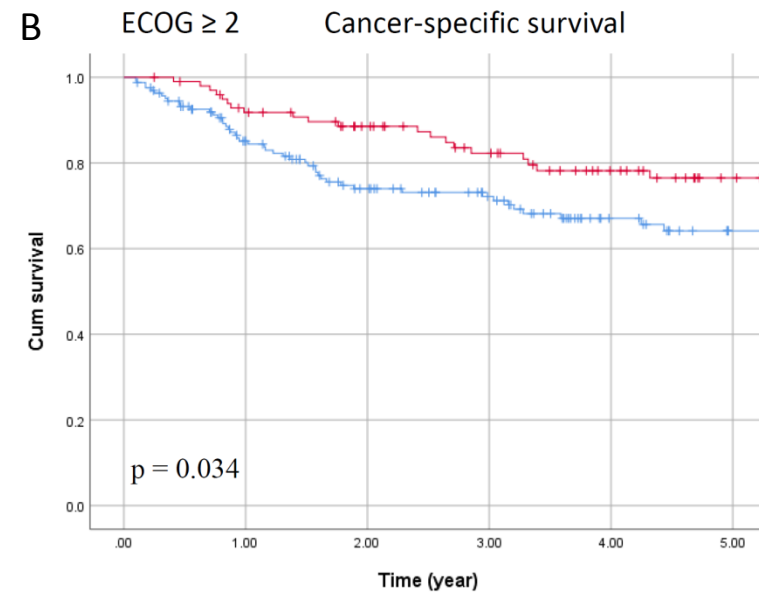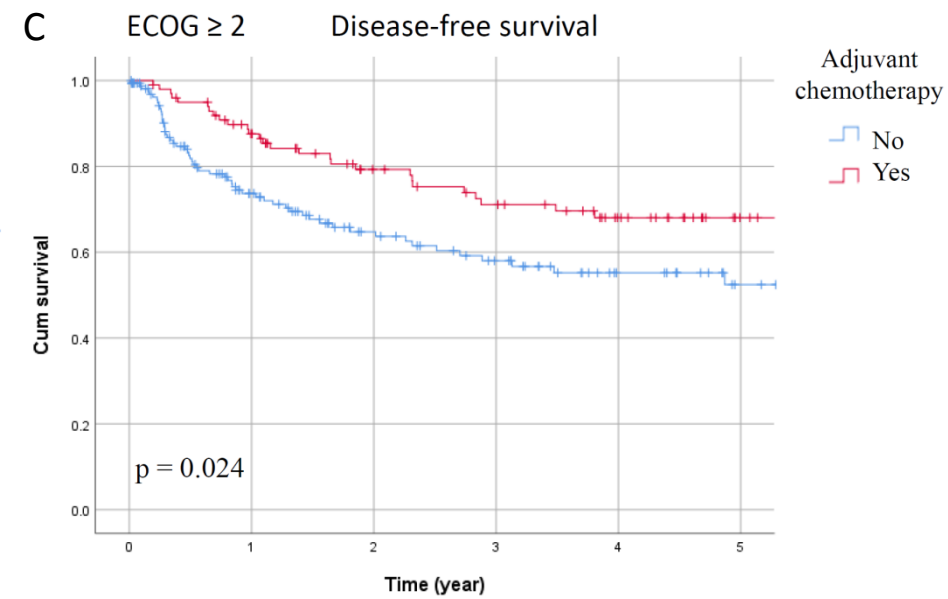

Supplement: Supplementary file 1 [file jpm-12-00226-s001.zip › jpm-1495908-supplementary/Figure S2.pdf]

Cancer-specific survival

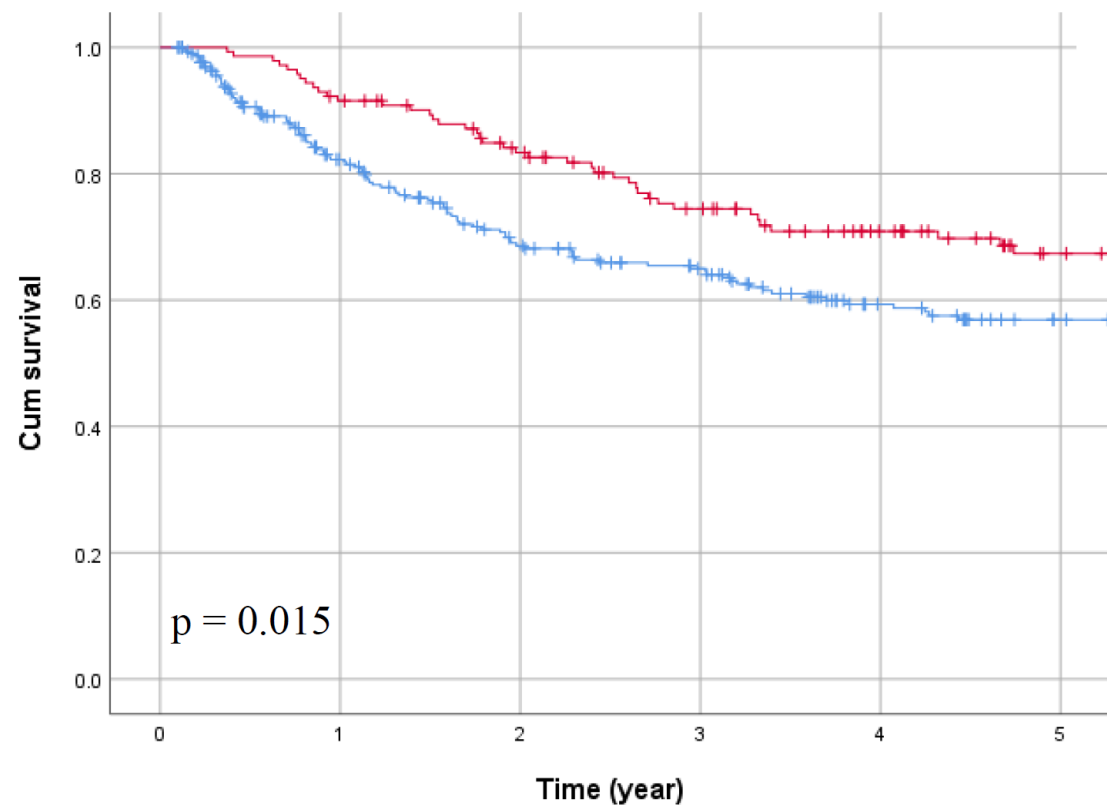

Disease-free survival

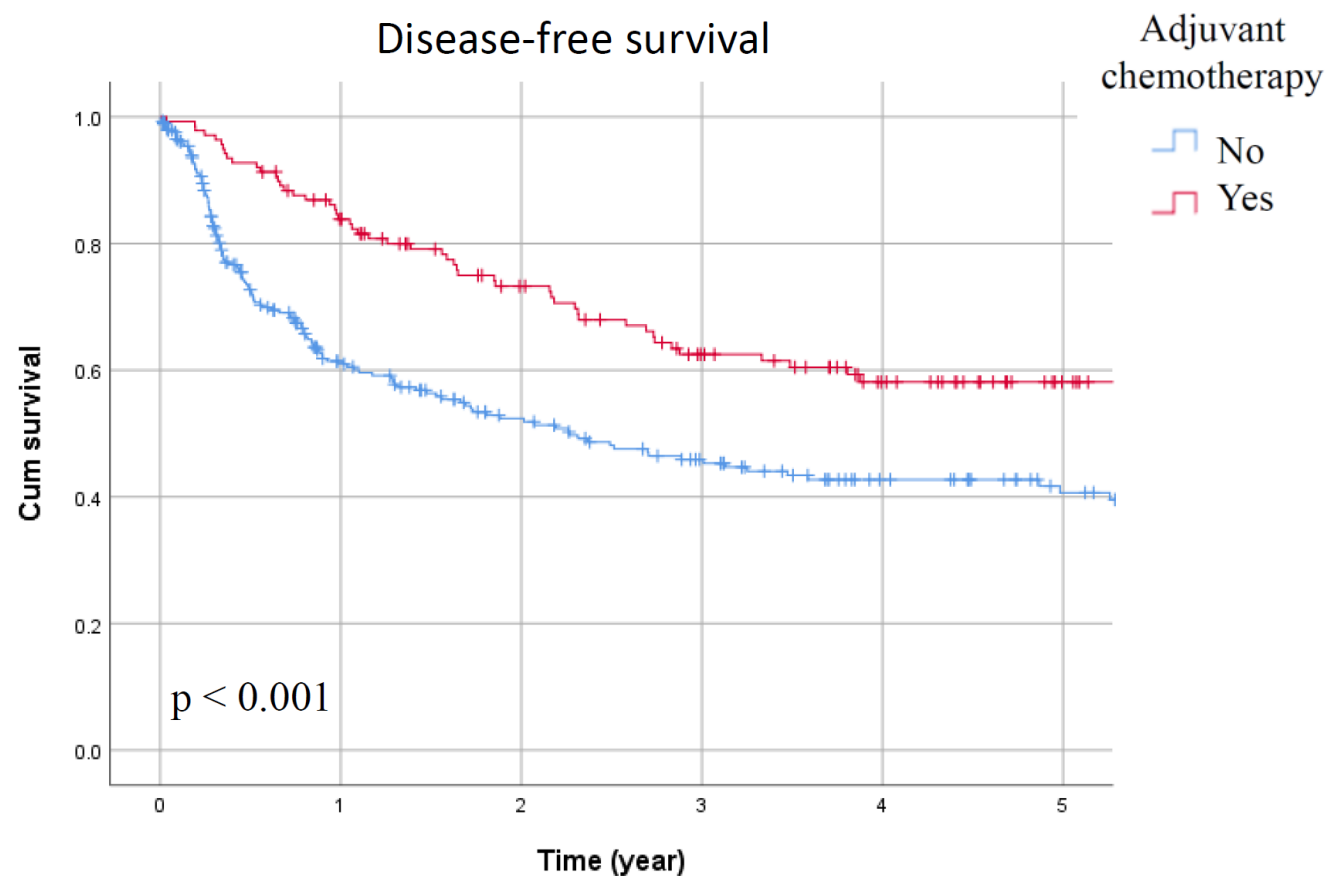

Supplement: Supplementary file 1 [file jpm-12-00226-s001.zip › jpm-1495908-supplementary/Figure S3.pdf]

Overall survival

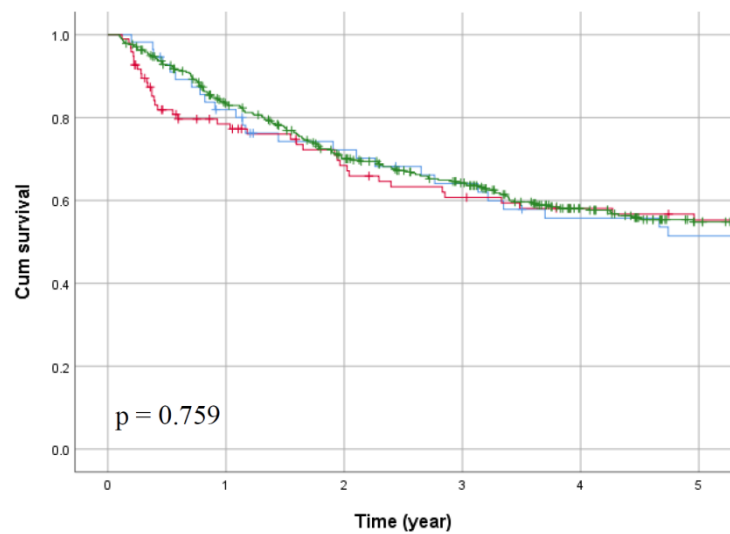

Cancer-specific survival

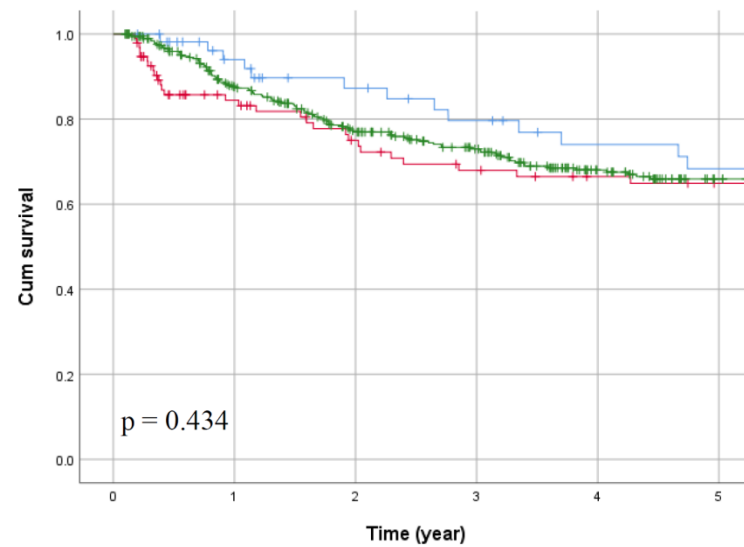

Disease-free survival

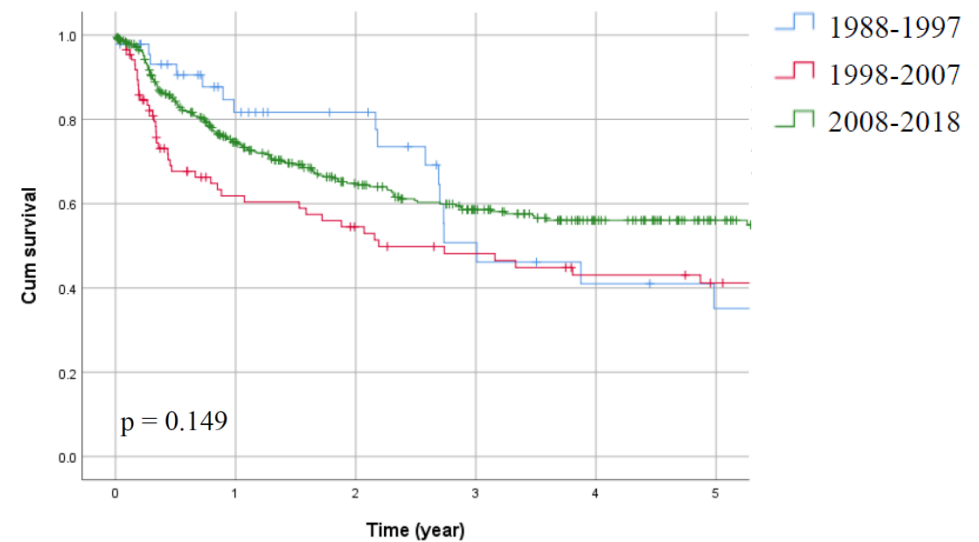

Year of diagnosis

- 1988-1997
- 1998-2007
- 2008-2018

Supplement: Supplementary file 1 [file jpm-12-00226-s001.zip › jpm-1495908-supplementary/Figure S4.pdf]
